# Psychometric Assessment of the Japanese Version of the Zurich Claudication Questionnaire (ZCQ): Reliability and Validity

**DOI:** 10.1371/journal.pone.0160183

**Published:** 2016-07-28

**Authors:** Nobuhiro Hara, Ko Matsudaira, Kazuhiro Masuda, Juichi Tohnosu, Katsushi Takeshita, Atsuki Kobayashi, Motoaki Murakami, Naohiro Kawamura, Kiyohumi Yamakawa, Sei Terayama, Satoshi Ogihara, Hiroo Shiono, Jiro Morii, Keiji Hayakawa, So Kato, Kozo Nakamura, Hiroyuki Oka, Takayuki Sawada, Kyoko Inuzuka, Norimasa Kikuchi

**Affiliations:** 1 Department of Orthopaedic Surgery, Musashino Red Cross Hospital, Musashino, Tokyo, Japan; 2 Department of Medical Research and Management for Musculoskeletal Pain, 22nd Century Medical & Research Center, Faculty of Medicine, The University of Tokyo, Bunkyo-ku, Tokyo, Japan; 3 Department of Orthopaedic Surgery, Tokyo Metropolitan Tama Medical Center, Fuchu, Tokyo, Japan; 4 Department of Orthopaedic Surgery, Kanto Rosai Hospital, Kawasaki, Kanagawa, Japan; 5 Department of Orthopaedic Surgery, Jichi Medical University, Shimotsuke, Tochigi, Japan; 6 Department of Orthopaedic Surgery, Tokyo Metropolitan Bokutoh Hospital, Sumida-ku, Tokyo, Japan; 7 Department of Orthopaedic Surgery, Toranomon Hospital, Minato-ku, Tokyo, Japan; 8 Department of Spine and Orthopaedic Surgery, Japanese Red Cross Medical Center, Shibuya-ku, Tokyo, Japan; 9 Department of Orthopaedic Surgery, Tokyo Metropolitan Komagome Hospital, Bunkyo-ku, Tokyo, Japan; 10 Department of Orthopaedic Surgery, Sangubashi Spine Surgery Hospital, Shibuya-ku, Tokyo, Japan; 11 Department of Orthopaedic Surgery, Sagamihara National Hospital, Sagamihara, Kanagawa, Japan; 12 Department of Orthopaedic Surgery, Sanraku Hospital, Chiyoda-ku, Tokyo, Japan; 13 Department of Orthopaedic Surgery, The University of Tokyo, Bunkyo-ku, Tokyo, Japan; 14 National Rehabilitation Center for Persons with Disabilities, Tokorozawa, Saitama, Japan; 15 Clinical Study Support, Inc., Nagoya, Aichi, Japan; Universita degli Studi di Palermo, ITALY

## Abstract

**Purpose:**

The Zurich Claudication Questionnaire (ZCQ) is a self-administered measure to evaluate symptom severity, physical function, and surgery satisfaction in lumbar spinal stenosis (LSS). The purpose of this study is to assess the psychometric properties of the Japanese ZCQ in LSS patients.

**Methods:**

LSS patients who are scheduled to undergo surgery were recruited from 12 facilities. Responses to several questionnaires, including the Japanese ZCQ; the visual analogue scale (VAS) to evaluate the degree of pain in the buttocks/legs, numbness in the buttocks/legs, and low back pain; the Oswestry Disability Index (ODI); and the SF-36v2, were collected before surgery and again 3 months after surgery (the post-surgery ZCQ was administered twice for test-retest reliability). For reliability, test-retest reliability was evaluated using the intra-class coefficient (ICC) and internal consistency was evaluated using Cronbach’s alpha coefficient. Concurrent validity was assessed using Spearman’s correlation coefficients between the Japanese ZCQ and other questionnaires. Effect size (ES) and standard response mean were calculated for responsiveness. All analyses were performed individually for the Japanese ZCQ symptom, function, and satisfaction domains.

**Results:**

Data from 180 LSS patients were used in this analysis. The ICCs were 0.81, 0.89, and 0.88 and Cronbach’s alpha coefficients were 0.78, 0.84, and 0.92 for the Japanese ZCQ symptom, function, and satisfaction domains, respectively. Regarding the concurrent validity, strong correlations (±0.5) were demonstrated between the Japanese ZCQ domains and the VAS leg pain, ODI, and SF-36v2 physical functioning or bodily pain, whereas correlations were approximately 0.3 in scales measuring other symptoms that are less related to symptom, function, or satisfaction domains. ESs showed high values for the ZCQ symptom and function domains (-1.73 for both).

**Conclusions:**

These psychometric assessments demonstrate that the Japanese ZCQ is a psychometrically reliable and valid measure in LSS. The Japanese ZCQ can evaluate both multi-dimensional aspects and the level of surgery satisfaction.

## Introduction

Lumbar spinal stenosis (LSS) is a degenerative disorder that is characterized by a narrowing of the lumbar spinal canal, which entraps and compresses intraspinal vascular and nerve structures [1]. LSS results in neurological symptoms in the lower extremities, such as leg pain/numbness and gait disturbance, that dramatically deteriorate the patients’ quality of life [2–4]. Conservative therapy is the primary treatment for LSS, and surgery is considered for LSS patients who do not improve [5]. Because pain or numbness is the primary complaint in LSS, the patient outcome measures have an important role in evaluating the treatment outcome.

Various outcome measures, such as the visual analogue scale (VAS) and Oswestry Disability Index (ODI) [6, 7], are used in research on LSS patients, but these measures are not disease specific. The Zurich Claudication Questionnaire (ZCQ), which is also known as both the Swiss Spinal Stenosis Measure and the Brigham Spinal Stenosis Questionnaire, was developed as a self-administered measure to assess symptom severity, physical function, and surgery satisfaction in LSS patients [8]. The questionnaire consists of three domains and uses a Likert-type scale. It includes 7 items for symptom severity with scores of 1 to 5, 5 items for functional disability with scores of 1 to 4, and 6 items for treatment satisfaction with score of 1 to 4. Higher scores indicate more severe LSS. The ZCQ demonstrates good validity and reliability in patients with LSS and is recommended as one of the appropriate methods for evaluating LSS treatment outcomes [9]. The ZCQ has been used worldwide in many studies on LSS [10–12].

To allow the use of the ZCQ in Japan, the English version was translated and linguistically validated as the Japanese ZCQ [13] following international guidelines [14, 15], but the psychometric validation has not yet been conducted.

The purpose of this study is to assess the psychometric properties of the Japanese ZCQ in LSS patients.

## Materials and Methods

The study was approved by the Ethics Committee at the University of Tokyo. All patients who were enrolled in the study had provided written informed consent.

### Participants

LSS patients between 20 and 85 years of age who were scheduled to undergo surgery were recruited. The inclusion criteria were as follows: 1) the presence of neurogenic intermittent claudication caused by numbness and/or pain in the lower limbs and 2) magnetic resonance imaging-confirmed symptomatic LSS that might explain the patient’s symptoms. The exclusion criteria were as follows: 1) a positive straight leg raising test result (sciatic pain at < 70 degrees of leg elevation), which indicates that the pain is likely to be due to lumbar disc herniation; 2) presentation with lower-extremity peripheral arterial disease; 3) a history of spinal surgery; 4) complications causing disorders that interfere with gait, such as myelopathy; 5) peripheral neuropathy, such as diabetic neuropathy of the leg; or 6) disorders that potentially hinder gait other than LSS (e.g., rheumatoid arthritis). The study was conducted in 2010 at the University of Tokyo and 11 affiliated facilities, all of which are located in or near Tokyo.

### Measures

Questionnaires were administered a maximum of 3 times: 1) before surgery, 2) 3 months after surgery, and 3) a few weeks after the second questionnaire if symptoms had not changed. The questionnaires administered before and 3 months after surgery included the Japanese versions of the ZCQ, ODI [6, 7], VAS for back/leg pain and leg numbness, and the SF-36 Ver.2 (SF-36v2). The ODI is a principal, condition-specific outcome measure used to assess disability from spinal disorders, particularly low back pain (LBP). The ODI consists of 10 items: pain intensity, personal care, lifting, walking, sitting, standing, sleeping, sex life, social life, and traveling. Each item is scored on a 6-point Likert-type scale with scores ranging from 0 to 5, and a higher score indicates a more severe disability. The reliability and validity of the Japanese version of the ODI were previously confirmed [16].

The degree of pain associated with buttock/leg pain or LBP was measured using a VAS covering the week prior to the relevant visit. We included three items: the degree of pain in the buttocks/legs, the degree of numbness in the buttocks/legs, and the degree of LBP. The scale ranged from 0 (no pain at all) to 10 (the worst pain/numbness imaginable).

The SF-36v2 is a questionnaire containing 36 items to assess the general health-related quality of life (QOL) [17]. The items are categorized into 8 domains: physical function, role limitations-physical, vitality, general health perception, bodily pain, social function, role limitations-emotional, and mental health. Each domain is scored from 0 to 100, with a higher score indicating a better QOL. A Japanese version of the SF-36v2, which has demonstrated good reliability and validity, was used in this study [18, 19].

### Statistical analysis

Demographic and clinical characteristics of the participants were analysed descriptively. The Japanese ZCQ domain scores were summarized to examine missing data and the distribution. The scoring for each domain was carried out in the same way it would be for the English version of the ZCQ [8].

Psychometric properties of the Japanese ZCQ were assessed by evaluating reliability and validity. Responsiveness was also assessed. Reliability was evaluated by test-retest reliability and internal consistency. For test-retest reliability, the extent of agreement between two time points was examined using the intra-class correlation coefficient (ICC) in patients with stable symptoms after spine surgery. The coefficient ranged from 0 to 1, with a higher value showing increased reliability. A coefficient greater than or equal to 0.7 was considered sufficient to determine test-retest reliability [20].

For internal consistency, the homogeneity of the items within the domain was evaluated using Cronbach’s alpha coefficients of the pre-surgery responses for the symptom and function domains and of the post-surgery responses for the satisfaction domain. A Cronbach’s alpha of 0.7 or higher was considered acceptable for internal consistency, while a score above 0.8 was good and above 0.9 was excellent [21].

For concurrent validity, the degree of correlation with the external criteria (ODI, VAS, and SF-36v2) was assessed using Spearman’s correlation coefficient of the pre-surgery responses for the symptom and function domains and of the post-surgery responses for the satisfaction domain. Scales measuring similar concepts were expected to show a moderate to strong correlation, while those measuring different concepts were expected to show a weak correlation. For example, the VASs of pain in the buttocks/legs and numbness in the buttocks/legs were expected to correlate strongly with the Japanese ZCQ symptom domain, the ODI with the ZCQ function domain, and the SF-36v2 social functioning or vitality with the ZCQ satisfaction domain. The correlation coefficient was interpreted as follows: ±0.1 was considered weak, ±0.3 was considered moderate, and ±0.5 was considered to be a strong correlation [21].

Responsiveness was evaluated by the effect sizes (ESs) and standard response means (SRMs). The ES was obtained by calculating the mean change in scores from before to 3 months after surgery divided by the standard deviation of the pre-surgery score. An ES of 0.2 was considered small, 0.5 was moderate and 0.8 was large, following the guidelines proposed by Cohen [22]. The SRM was obtained by the mean change in scores from before to 3 months after surgery divided by the standard deviation of the mean change. The higher the ES or SRM, the greater was the level of sensitivity to detect change.

All statistical tests were two sided with a significance level of 5%. All analyses were performed using SAS release 9.3 (SAS Institute, Cary, NC, USA).

## Results

### Patient characteristics

A total of 195 participants were recruited for this study. Of those, 180 took part in the first questionnaire administration before surgery, and 135 provided answers after surgery. Demographic and clinical characteristics of the recruited patients at pre-surgery are summarized in Table 1. The mean (standard deviation, SD) age was 68.2 (9.9) years, and 57.8% of the patients were male. The mean (SD) duration of LSS was approximately 3.7 (4.6) years. Types of symptom and surgery were evenly distributed. Approximately half the patients had spondylosis, followed by degenerative spondylolisthesis as the second most common diagnosis.

**Table 1:** Demographic and clinical characteristics of the lumber spinal stenosis patients (n = 195).

Characteristics	n (%) or mean (SD)
Age (years)	68.2 (9.9)
Sex	
Male	111 (57.8)
Female	81 (42.2)
Disease duration (months)	43.9 (55.7)
Types of symptom	
Nerve root	56 (29.3)
Cauda equine	65 (34.0)
Mixed	70 (36.7)
Surgery type	
Decompression only	92 (47.2)
Decompression and fusion	103 (52.8)
Types of lumber spinal stenosis	
Spondylosis	98 (50.8)
Degenerative spondylolisthesis	73 (37.8)
Degenerative scoliosis	18 (9.3)
Isthmic spondylolisthesis	4 (2.1)
Working status	
At work	65 (33.7)
Out of work	128 (66.3)
Smoking	
Smokers	47 (24.2)
Non-smokers	147 (75.8)

Values are n (%) or mean (SD).

Not all groups above total 195 because some of the characteristics had missing values.

The symptom, function, and satisfaction scores at pre- and post-surgery are summarized in Table 2. The mean and median were similar in all 3 domains at both time points. The mean or median scores of the symptom and function domains at 3 months after surgery are smaller than at pre-surgery.

**Table 2:** Distribution of the Zurich Claudication Questionnaire (ZCQ) subscales.

ZCQ Subscale	Pre-surgery (n = 180)*		3 months after surgery (n = 135)*	
	Mean (SD)	Median (minimum, maximum)	Mean (SD)	Median (minimum, maximum)
Symptom	3.41 ± 0.67	3.43 (1.57, 5.00)	2.25 ± 0.75	2.14 (1.00, 4.71)
Function	2.70 ± 0.57	2.80 (1.00, 3.75)	1.71 ± 0.66	1.50 (1.00, 3.60)
Satisfaction	–	–	1.97 ± 0.72	1.83 (1.00, 4.00)

Response of satisfaction was not obtained at pre-surgery. SD: Standard Deviation

*: Due to missing responses, domain scores for the symptom and function domains at 3 months after surgery could not be computed for one patient, and domain scores for the satisfaction domain at 3 months after surgery could not be computed for 2 patients.

### Reliability

To analyse test-retest reliability, 30 participants who underwent surgery and answered the Japanese ZCQ twice were selected. The ICCs for the Japanese ZCQ symptom, function, and satisfaction domains were 0.81, 0.89, and 0.88, respectively.

The internal consistency was evaluated using the data collected from patients who replied to the Japanese ZCQ at pre-surgery for the symptom and function domains and at post-surgery for the satisfaction domain. Cronbach’s alpha coefficients for the Japanese ZCQ symptom, function, and satisfaction domains were 0.78, 0.84, and 0.92, respectively.

### Validity

To assess the concurrent validity, the correlation coefficients between the 3 domains of the Japanese ZCQ and the external criteria (VAS, ODI, and SF-36v2) were calculated (Table 3). All 3 domains of the Japanese ZCQ correlated well with one another. Strong correlations with all 3 domains were observed in VAS leg pain by values of approximately 0.50–0.73, in ODI (0.63–0.75), and in physical functioning (-0.56–0.62) or bodily pain (-0.59–0.65) in the SF-36v2. Regarding the VAS, the coefficients of leg pain or leg numbness were generally higher than for the low back pain in all 3 Japanese ZCQ domains. With regard to each domain on the SF-36v2, strong correlations were observed between the Japanese ZCQ symptom domain and bodily pain or physical functioning; the ZCQ function domain and physical functioning, role limitations-physical, bodily pain, or role limitations-emotional; and the ZCQ satisfaction domain and all 8 SF-36v2 domains. Overall, all three Japanese ZCQ domains were correlated to all external criteria to a moderate to strong degree. However, all of the correlations between the Japanese symptom and function domains and the general health and mental health of the SF-36v2 were smaller: approximately 0.3.

**Table 3:** Spearman’s correlation coefficients (95% confidence interval) between the ZCQ and the ODI, VAS, and SF-36v2.

Measure	ZCQ Symptom	ZCQ Function	ZCQ Satisfaction
	(n = 180)	(n = 180)	(n = 135)
ZCQ symptom	–	0.63 (0.53, 0.71)	0.79 (0.72, 0.85)
ZCQ function	0.63 (0.53, 0.71)	–	0.73 (0.63, 0.80)
ZCQ satisfaction	–	–	–
VAS leg pain	0.50 (0.38, 0.60)	0.50 (0.38, 0.61)	0.73 (0.63, 0.80)
VAS leg numbness	0.58 (0.46, 0.67)	0.49 (0.36, 0.60)	0.67 (0.56, 0.76)
VAS low back pain	0.48 (0.35, 0.59)	0.42 (0.29, 0.54)	0.58 (0.44, 0.69)
ODI	0.63 (0.53, 0.71)	0.75 (0.67, 0.80)	0.74 (0.65, 0.80)
SF-36 physical functioning	-0.56 (-0.66, -0.45)	-0.62 (-0.70, -0.52)	-0.62 (-0.71, -0.50)
SF-36 role limitations-physical	-0.46 (-0.57, -0.34)	-0.51 (-0.61, -0.39)	-0.57 (-0.68, -0.44)
SF-36 bodily pain	-0.59 (-0.68, -0.48)	-0.63 (-0.71, -0.53)	-0.65 (-0.73, -0.53)
SF-36 social functioning	-0.44 (-0.55, -0.31)	-0.38 (-0.50, -0.24)	-0.61 (-0.71, -0.49)
SF-36 general health	-0.31 (-0.43, -0.17)	-0.28 (-0.41, -0.14)	-0.56 (-0.67, -0.43)
SF-36 vitality	-0.35 (-0.47, -0.21)	-0.41 (-0.52, -0.28)	-0.59 (-0.69, -0.46)
SF-36 role limitations-emotional	-0.46 (-0.57, -0.33)	-0.50 (-0.60, -0.38)	-0.50 (-0.61, -0.35)
SF-36 mental health	-0.31 (-0.44, -0.17)	-0.33 (-0.45, -0.19)	-0.54 (-0.65, -0.41)

Calculations of the ZCQ symptom and function domains were performed with the ZCQ responses at pre-surgery and those of the ZCQ satisfaction domain at 3 months after surgery.

95% Confidence interval is shown as lower and upper values. P < 0.0001 for all.

ZCQ, Zurich Claudication Questionnaire; ODI, Oswestry Disability Index; VAS, Visual Analogue Scale; SF-36, 36-Item Short-Form Health Survey Ver. 2.

### Responsiveness

To assess the responsiveness, the ESs between the Japanese ZCQ and external criteria (ODI, VAS, and SF-36v2) were calculated (Table 4). The ES was highest in the Japanese ZCQ function domain and symptom domain, followed by VAS leg pain, SF-36v2 bodily pain, VAS leg numbness, ODI, and VAS LBP, which were all above 0.8. Japanese ZCQ symptom and function domains had the two highest SRMs among the measures in this study (1.54 and 1.38, respectively).

**Table 4:** Responsiveness of outcome measures in the study.

Measure	Change at 3 months after surgery from before surgery		ES	SRM
	mean	SD		
ZCQ symptom	-1.16	0.75	-1.73	-1.54
ZCQ function	-1.01	0.71	-1.73	-1.38
ODI	-22.69	18.61	-1.22	-1.20
VAS leg pain	-3.86	3.41	-1.64	-1.20
VAS leg numbness	-3.37	2.98	-1.28	-1.20
VAS low back pain	-3.35	3.24	-1.15	-1.02
SF-36 physical functioning	12.98	16.89	0.74	0.83
SF-36 role limitations-physical	6.56	18.28	0.44	0.39
SF-36 bodily pain	12.52	13.34	1.44	0.91
SF-36 social functioning	6.14	18.61	0.40	0.36
SF-36 general health	4.40	8.08	0.45	0.52
SF-36 vitality	8.79	12.36	0.72	0.68
SF-36 role limitations-emotional	6.75	20.41	0.42	0.35
SF-36 mental health	7.38	14.03	0.54	0.52

Responsiveness of the ZCQ satisfaction domain was not calculated because a response on satisfaction was not obtained prior to surgery.

SD, Standard Deviation; ES, Effect Size; SRM, Standard Response Mean; ZCQ, Zurich Claudication Questionnaire; ODI, Oswestry Disability Index; VAS, Visual Analogue Scale; SF-36, 36-Item Short-Form Health Survey Ver. 2.

## Discussion

The Japanese ZCQ was translated and linguistically validated prior to this study [13]. As a next step for developing a valid and reliable measure, the psychometric properties of the ZCQ were assessed using the data collected from Japanese LSS patients. Based on the results of the current assessments, the Japanese ZCQ shows good validity and reliability. The responsiveness is also shown to be specific to LSS compared to other measures, such as the ODI or SF-36v2.

The ICCs for the 3 Japanese ZCQ domains (symptom, function, and surgery satisfaction) all satisfied the level of 0.7. The ICC was highest in the function domain and lowest in the symptom domain, but all were approximately 0.9. We set a satisfactory level of 0.7 for research use, but an ICC higher than 0.9 could be considered satisfactory for clinical practice in test-retest reliability [20, 21]. The ICC range was similar to other language versions, such as the original English (0.92), Norwegian (0.89–0.92), and simplified Chinese (0.91–0.95) versions [8, 23, 24].

Cronbach’s alpha coefficients showed good to excellent levels of internal consistency for all 3 domains, and these were approximately 0.9 in the function and surgery satisfaction domains. Although a Cronbach’s alpha of 0.7 is considered satisfactory for psychometric assessments, in clinical practice, values above 0.9 are considered suitable [20, 21]. Therefore, this measure is sufficient for application in clinical practice. Moreover, the range of Cronbach’s alpha coefficients was similar to that in other language versions, including the original English (0.84–0.89), Norwegian (0.94–0.96), Iranian (0.88), and simplified Chinese (0.86–0.91) versions [8, 23–25]. On the basis of these reliability results, the Japanese ZCQ showed sufficient reliability.

The concurrent validity assessment showed moderate to strong agreement with the external criteria (ODI, VAS, and 8 domains of the SF-36v2). Scales measuring similar concepts with each Japanese ZCQ domain showed moderate to strong correlations, such as the correlation between the ZCQ symptom domain and the bodily pain scale of the SF-36v2. Similar findings were also observed in the original English and simplified Chinese versions [8, 24]. All 3 Japanese ZCQ domains correlated strongly with one another, with the highest correlation observed between the symptom and satisfaction domains. By contrast, scales that measure different concepts showed smaller correlation coefficients, such as that between the Japanese ZCQ symptom and function domains and the general health scale of the SF-36v2. The correlation of the satisfaction domain was calculated using data obtained after surgery, when the mean changes in both symptom and function improved by a score of 1. This implies that patients in this study may have been satisfied when both their symptoms and function improved.

The Japanese ZCQ showed good responsiveness in both the symptom and function domains, and it showed better responsiveness than other measures, such as the ODI, which is commonly used to evaluate disability. The trend regarding responsiveness was similar to the results of other languages. For example, the ES and SRM of the symptom or function domain were between those of the original English (SRM = 1.48 and 1.6 in patients who were satisfied with surgery) and Norwegian (ES = 1.9 and 1.2) versions [23]. The current results showed that Japanese ZCQ symptom and function domains reflect changes in the post-operative condition of LSS in Japanese patients with a high degree of sensitivity. Because this measure can be used for multi-dimensional evaluations, is LSS specific, and has advantages in its simplicity and easy-to-answer format, the Japanese ZCQ may be useful for the elderly, who make up the majority of LSS patients. In addition, this questionnaire may enable better communication between the physician and the patient because sharing the responses of the patients evaluated in this study may enhance patient compliance with treatments.

There are several limitations of this study that should be mentioned. This study was carried out in Tokyo and its outlying areas. Because more than 10% of the Japanese population resides in this area, the study may over-represent the urban Japanese population. Therefore, we should consider the population of other suburban areas in Japan. Second, some physicians might have asked patients to complete the questionnaire in front of them and seen the responses the patients selected. This may have resulted in a bias in the responses being close to physicians’ expectations. Third, we did not assess known-group validity; i.e., score changes between the groups in terms of severity. However, we were able to show good responsiveness of the symptom and function domains in this study. Lastly, the presence of dynamic instability was not assessed in this study, although it plays an important role in the decision of treatment/surgery in a clinical setting. Patients’ responses to the questionnaire might have been influenced by the types of surgery/treatment; however, as this was a psychometric assessment study of the Japanese ZCQ, which will be widely used in patients with LSS, we consider that it is not a methodological problem to include patients with LSS regardless of the presence of dynamic instability.

## Conclusion

The current psychometric assessments have demonstrated that the Japanese ZCQ is psychometrically reliable and valid measure in LSS. The Japanese ZCQ, which includes symptom and function domains, can evaluate multi-dimensional aspects along with the level of surgery satisfaction.

## Supporting Information

S1 File Supporting information. Dataset of this study.
(XLS)
